# Supplementary material for: Characterization of indigenous chicken phenotypes in Liban Jawi District, Ethiopia: A qualitative and quantitative analysis
Source: PLoS One. 2025 Jan 10;20(1):e0307793. doi: 10.1371/journal.pone.0307793 (PMC11723647; doi:10.1371/journal.pone.0307793)
Supplement: S1 Table — (DOCX) [file pone.0307793.s001.docx]

S1 Traits of Chickens by Agro-Ecological Zones

| **SEX** | **PC** | **C type (A)** | **H type (B)** | **Er color (C)** | **Eye color (D)** | **S color (E)** | **skin col** | **F disturb** | **sh f** | **Agroecology** |
| --- | --- | --- | --- | --- | --- | --- | --- | --- | --- | --- |
| F | 9 | 3 | 2 | 2 | 1 | 5 | 1 | 1 | 1 | 1 |
| F | 11 | 1 | 1 | 1 | 1 | 1 | 1 | 1 | 1 | 1 |
| F | 1 | 1 | 1 | 2 | 1 | 6 | 1 | 1 | 2 | 1 |
| F | 6 | 3 | 1 | 4 | 1 | 5 | 1 | 1 | 2 | 1 |
| F | 9 | 1 | 1 | 1 | 2 | 6 | 1 | 1 | 1 | 1 |
| F | 3 | 1 | 1 | 4 | 2 | 6 | 1 | 1 | 2 | 1 |
| F | 3 | 1 | 2 | 1 | 2 | 5 | 1 | 1 | 1 | 1 |
| F | 9 | 4 | 2 | 2 | 2 | 6 | 1 | 1 | 2 | 1 |
| F | 9 | 1 | 1 | 4 | 1 | 6 | 1 | 1 | 1 | 1 |
| F | 9 | 1 | 2 | 4 | 2 | 6 | 1 | 1 | 2 | 1 |
| F | 5 | 1 | 2 | 4 | 2 | 5 | 1 | 1 | 1 | 1 |
| F | 1 | 3 | 1 | 1 | 1 | 6 | 1 | 1 | 2 | 1 |
| F | 5 | 1 | 1 | 2 | 2 | 5 | 1 | 1 | 2 | 1 |
| F | 9 | 1 | 2 | 2 | 1 | 6 | 1 | 1 | 1 | 1 |
| F | 1 | 4 | 2 | 1 | 2 | 6 | 1 | 1 | 2 | 1 |
| F | 9 | 1 | 2 | 1 | 1 | 6 | 1 | 1 | 2 | 1 |
| F | 7 | 1 | 2 | 1 | 1 | 6 | 2 | 1 | 2 | 1 |
| F | 5 | 1 | 2 | 1 | 2 | 5 | 2 | 1 | 1 | 1 |
| F | 7 | 4 | 1 | 1 | 2 | 5 | 2 | 1 | 2 | 1 |
| F | 8 | 1 | 1 | 2 | 1 | 5 | 2 | 1 | 1 | 1 |
| F | 13 | 1 | 1 | 2 | 2 | 2 | 2 | 1 | 2 | 1 |
| F | 9 | 3 | 2 | 1 | 1 | 6 | 2 | 1 | 1 | 1 |
| F | 13 | 1 | 2 | 1 | 2 | 6 | 2 | 1 | 2 | 1 |
| F | 9 | 1 | 2 | 1 | 1 | 6 | 2 | 1 | 1 | 1 |
| F | 14 | 3 | 2 | 1 | 2 | 2 | 2 | 1 | 2 | 1 |
| F | 3 | 3 | 1 | 1 | 2 | 6 | 2 | 1 | 1 | 1 |
| F | 5 | 1 | 1 | 1 | 1 | 6 | 2 | 1 | 2 | 1 |
| F | 5 | 1 | 1 | 1 | 1 | 1 | 2 | 1 | 2 | 1 |
| F | 6 | 1 | 2 | 1 | 2 | 6 | 2 | 1 | 1 | 1 |
| F | 6 | 1 | 2 | 2 | 1 | 6 | 1 | 1 | 1 | 1 |
| F | 11 | 1 | 2 | 2 | 2 | 6 | 1 | 1 | 1 | 1 |
| F | 5 | 4 | 1 | 1 | 1 | 1 | 1 | 1 | 1 | 1 |
| F | 9 | 1 | 1 | 1 | 2 | 6 | 1 | 1 | 1 | 1 |
| F | 6 | 1 | 2 | 2 | 1 | 6 | 1 | 1 | 1 | 1 |
| F | 6 | 1 | 1 | 1 | 2 | 6 | 1 | 1 | 1 | 1 |
| F | 9 | 1 | 2 | 1 | 2 | 1 | 1 | 1 | 1 | 1 |
| F | 10 | 1 | 1 | 1 | 1 | 6 | 2 | 1 | 1 | 1 |
| F | 9 | 3 | 2 | 1 | 1 | 1 | 2 | 1 | 1 | 1 |
| F | 9 | 1 | 1 | 2 | 2 | 5 | 1 | 1 | 1 | 1 |
| F | 8 | 3 | 2 | 4 | 2 | 5 | 1 | 1 | 1 | 1 |
| F | 9 | 1 | 2 | 1 | 2 | 6 | 1 | 1 | 1 | 1 |
| F | 9 | 1 | 1 | 1 | 2 | 1 | 2 | 1 | 1 | 1 |
| F | 2 | 1 | 1 | 2 | 1 | 5 | 2 | 1 | 1 | 1 |
| F | 9 | 3 | 2 | 1 | 1 | 6 | 1 | 1 | 1 | 1 |
| F | 9 | 4 | 2 | 1 | 1 | 6 | 2 | 1 | 2 | 1 |
| F | 5 | 1 | 2 | 1 | 1 | 6 | 1 | 1 | 1 | 1 |
| F | 9 | 1 | 1 | 1 | 1 | 1 | 1 | 1 | 2 | 1 |
| F | 5 | 1 | 1 | 2 | 1 | 6 | 1 | 1 | 1 | 1 |
| F | 2 | 1 | 2 | 1 | 3 | 2 | 1 | 1 | 1 | 1 |
| F | 9 | 1 | 1 | 4 | 1 | 6 | 1 | 1 | 1 | 1 |
| F | 9 | 1 | 2 | 4 | 1 | 6 | 1 | 1 | 1 | 1 |
| F | 9 | 3 | 2 | 1 | 1 | 6 | 1 | 1 | 1 | 1 |
| F | 4 | 3 | 1 | 3 | 2 | 5 | 1 | 1 | 2 | 1 |
| F | 8 | 1 | 2 | 1 | 2 | 5 | 1 | 1 | 1 | 1 |
| F | 5 | 3 | 1 | 3 | 2 | 6 | 1 | 1 | 1 | 1 |
| F | 5 | 1 | 2 | 1 | 1 | 1 | 1 | 1 | 1 | 2 |
| F | 9 | 1 | 2 | 4 | 2 | 6 | 2 | 1 | 2 | 2 |
| F | 5 | 3 | 2 | 1 | 2 | 1 | 2 | 1 | 1 | 2 |
| F | 9 | 1 | 1 | 4 | 2 | 6 | 2 | 1 | 1 | 2 |
| F | 5 | 1 | 2 | 1 | 1 | 5 | 2 | 1 | 1 | 2 |
| F | 3 | 4 | 1 | 2 | 1 | 5 | 2 | 1 | 1 | 2 |
| F | 5 | 1 | 2 | 1 | 1 | 6 | 2 | 1 | 1 | 2 |
| F | 5 | 3 | 2 | 1 | 1 | 1 | 2 | 1 | 1 | 2 |
| F | 5 | 3 | 1 | 1 | 2 | 6 | 2 | 1 | 1 | 2 |
| F | 9 | 1 | 2 | 1 | 2 | 6 | 1 | 1 | 1 | 2 |
| F | 9 | 4 | 2 | 1 | 2 | 1 | 1 | 1 | 1 | 2 |
| F | 5 | 3 | 1 | 1 | 1 | 6 | 1 | 1 | 1 | 2 |
| F | 13 | 1 | 2 | 1 | 1 | 6 | 1 | 1 | 2 | 2 |
| F | 12 | 4 | 1 | 2 | 1 | 6 | 1 | 1 | 1 | 2 |
| F | 5 | 3 | 1 | 1 | 1 | 6 | 1 | 1 | 1 | 2 |
| F | 5 | 1 | 2 | 2 | 1 | 6 | 1 | 1 | 1 | 2 |
| F | 5 | 3 | 1 | 2 | 1 | 5 | 2 | 1 | 1 | 2 |
| F | 5 | 1 | 2 | 4 | 2 | 6 | 2 | 1 | 1 | 2 |
| F | 9 | 1 | 2 | 4 | 2 | 5 | 2 | 1 | 2 | 2 |
| F | 5 | 1 | 1 | 4 | 2 | 1 | 2 | 1 | 2 | 2 |
| F | 9 | 1 | 1 | 4 | 1 | 6 | 2 | 1 | 1 | 2 |
| F | 5 | 1 | 2 | 2 | 1 | 6 | 2 | 1 | 1 | 2 |
| F | 1 | 1 | 2 | 1 | 1 | 1 | 2 | 1 | 1 | 2 |
| F | 5 | 4 | 2 | 1 | 2 | 6 | 2 | 1 | 1 | 2 |
| F | 6 | 3 | 2 | 1 | 2 | 5 | 1 | 1 | 1 | 2 |
| F | 5 | 1 | 1 | 1 | 2 | 6 | 1 | 1 | 1 | 2 |
| F | 11 | 1 | 1 | 2 | 2 | 6 | 1 | 1 | 1 | 2 |
| F | 8 | 3 | 2 | 2 | 2 | 6 | 1 | 1 | 1 | 2 |
| F | 5 | 1 | 2 | 2 | 2 | 1 | 1 | 1 | 1 | 2 |
| F | 1 | 1 | 1 | 1 | 2 | 1 | 1 | 1 | 1 | 2 |
| F | 4 | 1 | 2 | 1 | 1 | 4 | 1 | 1 | 1 | 2 |
| F | 5 | 1 | 1 | 2 | 1 | 5 | 1 | 1 | 1 | 2 |
| F | 1 | 1 | 2 | 4 | 1 | 5 | 1 | 1 | 1 | 2 |
| F | 5 | 1 | 1 | 4 | 1 | 6 | 1 | 1 | 1 | 2 |
| F | 9 | 1 | 2 | 2 | 1 | 6 | 1 | 1 | 1 | 2 |
| F | 5 | 1 | 1 | 3 | 1 | 5 | 1 | 1 | 1 | 3 |
| F | 3 | 1 | 2 | 4 | 1 | 6 | 1 | 1 | 1 | 3 |
| F | 1 | 3 | 1 | 3 | 2 | 6 | 1 | 1 | 1 | 3 |
| F | 10 | 1 | 2 | 1 | 2 | 1 | 1 | 1 | 1 | 3 |
| F | 11 | 1 | 1 | 1 | 2 | 6 | 1 | 1 | 1 | 3 |
| F | 10 | 3 | 2 | 1 | 2 | 1 | 2 | 1 | 1 | 3 |
| F | 10 | 1 | 1 | 3 | 1 | 6 | 2 | 1 | 1 | 3 |
| F | 5 | 1 | 2 | 2 | 2 | 6 | 2 | 1 | 1 | 3 |
| F | 10 | 2 | 2 | 1 | 3 | 5 | 2 | 1 | 1 | 3 |
| F | 10 | 2 | 2 | 2 | 3 | 5 | 2 | 1 | 2 | 3 |
| F | 6 | 3 | 2 | 2 | 1 | 6 | 2 | 1 | 2 | 3 |
| F | 10 | 4 | 2 | 2 | 3 | 5 | 2 | 1 | 2 | 3 |
| F | 8 | 2 | 2 | 1 | 1 | 6 | 2 | 1 | 2 | 3 |
| F | 5 | 3 | 2 | 1 | 2 | 1 | 2 | 1 | 1 | 3 |
| F | 10 | 1 | 2 | 2 | 2 | 1 | 2 | 1 | 1 | 3 |
| F | 9 | 2 | 2 | 1 | 3 | 2 | 1 | 1 | 1 | 3 |
| F | 10 | 1 | 1 | 1 | 2 | 1 | 1 | 1 | 1 | 3 |
| F | 2 | 2 | 1 | 2 | 3 | 6 | 1 | 1 | 1 | 3 |
| F | 10 | 2 | 1 | 1 | 2 | 5 | 1 | 1 | 1 | 3 |
| F | 1 | 1 | 2 | 2 | 3 | 6 | 1 | 1 | 1 | 3 |
| F | 10 | 3 | 1 | 1 | 2 | 5 | 1 | 1 | 1 | 3 |
| F | 5 | 2 | 2 | 1 | 3 | 6 | 1 | 1 | 1 | 3 |
| F | 10 | 1 | 1 | 2 | 2 | 6 | 1 | 1 | 2 | 3 |
| F | 4 | 2 | 2 | 1 | 3 | 3 | 1 | 1 | 2 | 3 |
| F | 10 | 2 | 1 | 2 | 2 | 6 | 2 | 1 | 2 | 3 |
| F | 10 | 2 | 2 | 1 | 3 | 5 | 2 | 1 | 1 | 3 |
| F | 1 | 1 | 1 | 2 | 2 | 3 | 1 | 1 | 1 | 3 |
| F | 10 | 3 | 2 | 1 | 3 | 6 | 2 | 1 | 1 | 3 |
| F | 4 | 1 | 2 | 1 | 1 | 3 | 2 | 1 | 1 | 3 |
| F | 10 | 1 | 2 | 2 | 2 | 1 | 2 | 1 | 1 | 3 |
| F | 10 | 1 | 2 | 1 | 3 | 5 | 2 | 1 | 1 | 3 |
| F | 9 | 1 | 2 | 4 | 1 | 6 | 2 | 1 | 1 | 3 |
| F | 10 | 1 | 2 | 4 | 3 | 6 | 2 | 1 | 1 | 3 |
| F | 5 | 1 | 2 | 1 | 2 | 5 | 2 | 1 | 1 | 3 |
| F | 10 | 1 | 1 | 2 | 3 | 6 | 2 | 1 | 1 | 3 |
| M | 11 | 4 | 1 | 1 | 3 | 1 | 2 | 1 | 1 | 1 |
| M | 3 | 1 | 1 | 1 | 2 | 6 | 1 | 1 | 1 | 1 |
| M | 10 | 3 | 1 | 1 | 2 | 6 | 1 | 1 | 1 | 1 |
| M | 4 | 1 | 2 | 2 | 1 | 6 | 1 | 1 | 1 | 1 |
| M | 10 | 1 | 1 | 2 | 1 | 3 | 1 | 1 | 2 | 1 |
| M | 9 | 1 | 2 | 2 | 3 | 4 | 1 | 1 | 2 | 1 |
| M | 10 | 1 | 1 | 2 | 1 | 6 | 1 | 1 | 2 | 1 |
| M | 10 | 1 | 2 | 1 | 1 | 6 | 1 | 1 | 1 | 1 |
| M | 12 | 1 | 1 | 1 | 1 | 6 | 1 | 1 | 1 | 1 |
| M | 10 | 1 | 1 | 3 | 3 | 1 | 1 | 1 | 1 | 1 |
| M | 12 | 1 | 2 | 1 | 3 | 6 | 1 | 1 | 1 | 1 |
| M | 12 | 4 | 1 | 1 | 2 | 1 | 1 | 1 | 1 | 1 |
| M | 12 | 1 | 2 | 1 | 2 | 6 | 1 | 1 | 1 | 1 |
| M | 9 | 1 | 1 | 1 | 2 | 6 | 1 | 1 | 1 | 1 |
| M | 12 | 1 | 2 | 1 | 3 | 5 | 2 | 1 | 1 | 1 |
| M | 9 | 1 | 2 | 2 | 3 | 5 | 2 | 1 | 1 | 1 |
| M | 12 | 1 | 2 | 2 | 3 | 6 | 2 | 1 | 2 | 1 |
| M | 9 | 1 | 2 | 1 | 3 | 6 | 1 | 1 | 1 | 1 |
| M | 12 | 1 | 2 | 2 | 1 | 6 | 1 | 1 | 1 | 1 |
| M | 1 | 1 | 2 | 2 | 3 | 5 | 1 | 1 | 1 | 1 |
| M | 12 | 1 | 1 | 1 | 1 | 6 | 1 | 1 | 1 | 1 |
| M | 1 | 1 | 2 | 1 | 3 | 5 | 1 | 1 | 1 | 1 |
| M | 12 | 4 | 1 | 1 | 1 | 6 | 1 | 1 | 2 | 1 |
| M | 12 | 1 | 1 | 2 | 2 | 1 | 1 | 1 | 1 | 1 |
| M | 12 | 1 | 1 | 1 | 2 | 6 | 1 | 1 | 1 | 1 |
| M | 4 | 1 | 1 | 1 | 1 | 1 | 2 | 1 | 1 | 1 |
| M | 12 | 1 | 2 | 2 | 1 | 5 | 1 | 1 | 1 | 1 |
| M | 12 | 1 | 2 | 2 | 2 | 6 | 1 | 1 | 1 | 1 |
| M | 7 | 1 | 1 | 1 | 3 | 6 | 1 | 1 | 1 | 1 |
| M | 12 | 1 | 2 | 2 | 2 | 1 | 1 | 1 | 1 | 1 |
| M | 12 | 1 | 2 | 1 | 2 | 1 | 1 | 1 | 1 | 1 |
| M | 13 | 3 | 1 | 2 | 2 | 6 | 2 | 1 | 1 | 1 |
| M | 12 | 4 | 2 | 2 | 3 | 2 | 1 | 1 | 2 | 1 |
| M | 10 | 1 | 2 | 1 | 3 | 6 | 1 | 1 | 1 | 1 |
| M | 10 | 1 | 1 | 1 | 3 | 2 | 1 | 1 | 2 | 1 |
| M | 12 | 1 | 1 | 1 | 3 | 6 | 1 | 1 | 2 | 1 |
| M | 5 | 1 | 1 | 1 | 3 | 5 | 1 | 1 | 1 | 1 |
| M | 12 | 3 | 1 | 2 | 3 | 5 | 1 | 1 | 1 | 1 |
| M | 12 | 1 | 2 | 2 | 1 | 6 | 1 | 1 | 1 | 1 |
| M | 12 | 1 | 1 | 2 | 1 | 6 | 1 | 1 | 1 | 1 |
| M | 5 | 3 | 2 | 2 | 1 | 1 | 1 | 1 | 2 | 1 |
| M | 12 | 1 | 2 | 1 | 2 | 6 | 1 | 1 | 1 | 1 |
| M | 5 | 1 | 2 | 1 | 2 | 1 | 1 | 1 | 2 | 1 |
| M | 12 | 1 | 2 | 2 | 2 | 6 | 1 | 1 | 2 | 1 |
| M | 12 | 1 | 2 | 2 | 2 | 5 | 1 | 1 | 1 | 2 |
| M | 12 | 1 | 1 | 1 | 2 | 6 | 1 | 1 | 1 | 2 |
| M | 12 | 1 | 2 | 1 | 3 | 6 | 2 | 1 | 1 | 2 |
| M | 9 | 3 | 2 | 2 | 1 | 6 | 1 | 1 | 1 | 2 |
| M | 1 | 1 | 2 | 4 | 1 | 6 | 1 | 1 | 1 | 2 |
| M | 5 | 1 | 2 | 4 | 1 | 1 | 1 | 1 | 1 | 2 |
| M | 5 | 4 | 1 | 2 | 2 | 6 | 1 | 1 | 2 | 2 |
| M | 9 | 1 | 2 | 3 | 1 | 2 | 1 | 1 | 2 | 2 |
| M | 9 | 3 | 1 | 4 | 2 | 2 | 2 | 1 | 2 | 2 |
| M | 8 | 1 | 2 | 3 | 3 | 6 | 1 | 1 | 2 | 2 |
| M | 8 | 3 | 1 | 1 | 1 | 1 | 1 | 1 | 2 | 2 |
| M | 6 | 1 | 1 | 1 | 3 | 6 | 1 | 1 | 1 | 2 |
| M | 6 | 1 | 2 | 1 | 1 | 1 | 1 | 1 | 1 | 2 |
| M | 6 | 1 | 2 | 3 | 1 | 5 | 1 | 1 | 1 | 2 |
| M | 6 | 1 | 1 | 2 | 1 | 6 | 1 | 1 | 1 | 2 |
| M | 7 | 1 | 1 | 1 | 3 | 1 | 2 | 1 | 1 | 2 |
| M | 8 | 3 | 2 | 2 | 3 | 6 | 2 | 1 | 1 | 2 |
| M | 7 | 1 | 2 | 2 | 2 | 6 | 2 | 1 | 2 | 2 |
| M | 8 | 2 | 1 | 2 | 2 | 5 | 2 | 1 | 1 | 2 |
| M | 9 | 1 | 1 | 1 | 2 | 1 | 2 | 1 | 1 | 2 |
| M | 9 | 2 | 1 | 1 | 3 | 5 | 2 | 1 | 1 | 2 |
| M | 5 | 4 | 2 | 2 | 3 | 6 | 2 | 1 | 1 | 3 |
| M | 5 | 1 | 2 | 1 | 2 | 6 | 2 | 1 | 1 | 3 |
| M | 5 | 1 | 1 | 1 | 2 | 6 | 2 | 1 | 1 | 3 |
| M | 4 | 2 | 2 | 2 | 2 | 6 | 2 | 1 | 2 | 3 |
| M | 5 | 1 | 2 | 1 | 1 | 5 | 1 | 1 | 1 | 3 |
| M | 3 | 2 | 2 | 2 | 1 | 5 | 1 | 1 | 1 | 3 |
| M | 8 | 2 | 2 | 1 | 2 | 6 | 1 | 1 | 1 | 3 |
| M | 3 | 2 | 1 | 1 | 1 | 5 | 1 | 1 | 1 | 3 |
| M | 11 | 3 | 2 | 2 | 1 | 6 | 2 | 1 | 1 | 3 |
| M | 9 | 2 | 1 | 1 | 3 | 1 | 1 | 1 | 1 | 3 |
| M | 9 | 2 | 2 | 2 | 3 | 5 | 1 | 1 | 2 | 3 |
| M | 5 | 1 | 2 | 1 | 1 | 1 | 1 | 1 | 1 | 3 |
| M | 5 | 1 | 2 | 2 | 3 | 6 | 1 | 1 | 1 | 3 |
| M | 3 | 2 | 1 | 1 | 3 | 6 | 1 | 1 | 1 | 3 |
| M | 10 | 2 | 1 | 1 | 1 | 6 | 1 | 1 | 2 | 3 |
| M | 10 | 2 | 1 | 1 | 1 | 1 | 2 | 1 | 1 | 3 |
| M | 6 | 2 | 2 | 2 | 1 | 6 | 1 | 1 | 1 | 3 |
| M | 9 | 1 | 2 | 2 | 2 | 6 | 1 | 1 | 1 | 3 |
| M | 9 | 2 | 1 | 1 | 2 | 5 | 1 | 1 | 1 | 3 |
| M | 9 | 4 | 2 | 1 | 2 | 6 | 2 | 1 | 1 | 3 |

Keys:

**plumage color (PC)** (1=black, 2= black mottled, 3=Black-laced white, 4=Brown, 5=Brown mottled, 6=Dark brown, 7=Dark Brown mottled, 8=Greyish mixture, 9=Red, 10=Reddish brown, 11= Wheaten, 12= Wheaten mottled, 13=White , 14=White mottled , 15=White-laced black

**C type =(**Color Type) (1=cushion, 2= Pea, 3= rose, 4=single, 5= double)

**H Type (**Hue Type**)** (1=crest, 2= plain)

**ER color** (External Reflective Color) (1=red, 2=white, 3=yellow, 4=brown)

**E COLOR** (Emitted Color) (1= pearl, 2= Brown, 3= Orange, 4= red)

**S COLOR** (Structural Color) (E1.black, E2.Bluish black, E3.Green, E4.Green blue, E5.White, E6.Yellow
