# Supplementary material for: Characterization of indigenous chicken phenotypes in Liban Jawi District, Ethiopia: A qualitative and quantitative analysis
Source: PLoS One. 2025 Jan 10;20(1):e0307793. doi: 10.1371/journal.pone.0307793 (PMC11723647; doi:10.1371/journal.pone.0307793)
Supplement: Attachment — Submitted filename: Comments in highlight PONE-D-24-28488.pdf [file pone.0307793.s002.pdf]

PLOS ONE

Characterization of Indigenous Chicken Phenotypes in Liban Jawi District, Ethiopia: A Qualitative and Quantitative Analysis

--Manuscript Draft--

**Manuscript Number:**	PONE-D-24-28488
**Article Type:**	Research Article
**Full Title:**	Characterization of Indigenous Chicken Phenotypes in Liban Jawi District, Ethiopia: A Qualitative and Quantitative Analysis
**Short Title:**	Phenotypic Characterization of Indigenous Chickens in Liban Jaw district, Ethiopia
**Corresponding Author:**	Desalegn Begna, Ph.D<br>Policy Studies Institute, Ethiopia<br>Addis Ababa, ETHIOPIA
**Keywords:**	Quantitative, Indigenous, Chicken, Qualitative, Phenotypic
**Abstract:**	Indigenous chickens play a crucial role in the livelihoods of smallholder farmers in rural Ethiopia. This study aimed to phenotypically characterize indigenous chickens in the Liban Jawi district, focusing on phenotypic characteristics measurements. A total of 224 chickens were selected from a larger population of 2452 chickens in the study areas. The selected chickens were segregated by sex and agroecologies, and comprehensive phenotypic characteristics measurements were taken.<br>The analysis of qualitative traits revealed that the male chickens exhibited a plain head shape (54.7%), single combs (39.5%), red feather plumage (26.7%), yellow shanks (54.7%), red earlobes (51.6%), and white skin (68.6%). Among the female chickens, single combs (75.4%), red earlobes (51.4%), yellow shanks (50.1%), and brown mottled feathers (23.9%), with red (23.1%) and black (9.4%) feathers observed.<br>The average body weight of cocks was 1.46±0.02 kg, while hens were 1.21±0.01 kg. Cocks exhibited larger body dimensions, with average body lengths, body widths, shank lengths, shank circumferences, and wingspans measuring 38.05±0.26 cm, 22.20±0.20 cm, 8.76±0.11 cm, 3.58±0.05 cm, and 38.56±0.13 cm, respectively. Correspondingly, hens had an average of 31.55±0.33 cm in body length, 19.04±0.21 cm in body width, 6.66±0.05 cm in shank length, 3.36±0.03 cm in shank circumference, and 32.98±0.13 cm in wingspan. Particularly, shank length emerged as the best predictor of body weight for both hens (r=0.45) and cocks (r=0.44).<br>The study revealed significant variations in both qualitative and quantitative traits among the indigenous chicken population, suggesting the potential for improvement through selective breeding at the community level. These findings provide valuable insights into the phenotypic characterization of indigenous chickens in the study district, serving as a foundation for future breeding programs and conservation efforts aimed at enhancing productivity and preserving genetic diversity. Further molecular-level characterization of the indigenous chicken population is recommended to validate the current phenotypic results.
**Order of Authors:**	Desalegn Begna, Ph.D
	Teferi Bacha, Msc
	Shambel Boki, Msc
**Additional Information:**	
**Question**	**Response**
**Financial Disclosure**<br><br>Enter a financial disclosure statement that describes the sources of funding for the work included in this submission. Review the submission guidelines for detailed requirements. View published research	The author(s) received no specific funding for this work.

articles from *PLOS ONE* for specific examples.<br><br>This statement is required for submission and **will appear in the published article** if the submission is accepted. Please make sure it is accurate.<br><br>**Funded studies**<br>Enter a statement with the following details:<br>• Initials of the authors who received each award<br>• Grant numbers awarded to each author<br>• The full name of each funder<br>• URL of each funder website<br>• Did the sponsors or funders play any role in the study design, data collection and analysis, decision to publish, or preparation of the manuscript?<br><br>Did you receive funding for this work?	
**Competing Interests**<br><br>Use the instructions below to enter a competing interest statement for this submission. On behalf of all authors, disclose any competing interests that could be perceived to bias this work—acknowledging all financial support and any other relevant financial or non-financial competing interests.<br><br>This statement is required for submission and **will appear in the published article** if the submission is accepted. Please make sure it is accurate and that any funding sources listed in your Funding Information later in the submission form are also declared in your Financial Disclosure statement.<br><br>View published research articles from *PLOS ONE* for specific examples.	The authors have declared that no competing interests exist

**NO authors have competing interests**<br><br>Enter: *The authors have declared that no competing interests exist.*<br><br>**Authors with competing interests**<br><br>Enter competing interest details beginning with this statement:<br><br>*I have read the journal's policy and the authors of this manuscript have the following competing interests: [insert competing interests here]*<br><br>* typeset	
**Ethics Statement**<br><br>Enter an ethics statement for this submission. This statement is required if the study involved:<br><br>• Human participants<br>• Human specimens or tissue<br>• Vertebrate animals or cephalopods<br>• Vertebrate embryos or tissues<br>• Field research<br><br>Write "N/A" if the submission does not require an ethics statement.<br><br>General guidance is provided below. Consult the submission guidelines for detailed instructions. **Make sure that all information entered here is included in the Methods section of the manuscript.**	Not applicable

**Format for specific study types**<br><br>**Human Subject Research (involving human participants and/or tissue)**<br>• Give the name of the institutional review board or ethics committee that approved the study<br>• Include the approval number and/or a statement indicating approval of this research<br>• Indicate the form of consent obtained (written/oral) or the reason that consent was not obtained (e.g. the data were analyzed anonymously)<br><br>**Animal Research (involving vertebrate animals, embryos or tissues)**<br>• Provide the name of the Institutional Animal Care and Use Committee (IACUC) or other relevant ethics board that reviewed the study protocol, and indicate whether they approved this research or granted a formal waiver of ethical approval<br>• Include an approval number if one was obtained<br>• If the study involved *non-human primates*, add *additional details* about animal welfare and steps taken to ameliorate suffering<br>• If anesthesia, euthanasia, or any kind of animal sacrifice is part of the study, include briefly which substances and/or methods were applied<br><br>**Field Research**<br><br>Include the following details if this study involves the collection of plant, animal, or other materials from a natural setting:<br>• Field permit number<br>• Name of the institution or relevant body that granted permission	
**Data Availability**<br><br>Authors are required to make all data underlying the findings described fully available, without restriction, and from the time of publication. PLOS allows rare exceptions to address legal and ethical concerns. See the PLOS Data Policy and FAQ for detailed information.	Yes - all data are fully available without restriction

Powered by Editorial Manager® and ProduXion Manager® from Aries Systems Corporation

A Data Availability Statement describing where the data can be found is required at submission. Your answers to this question constitute the Data Availability Statement and **will be published in the article**, if accepted.<br><br>**Important:** Stating 'data available on request from the author' is not sufficient. If your data are only available upon request, select 'No' for the first question and explain your exceptional situation in the text box.<br><br>Do the authors confirm that all data underlying the findings described in their manuscript are fully available without restriction?	
**Describe where the data may be found in full sentences. If you are copying our sample text, replace any instances of XXX with the appropriate details.**<br><br>• If the data are **held or will be held in a public repository**, include URLs, accession numbers or DOIs. If this information will only be available after acceptance, indicate this by ticking the box below. For example: *All XXX files are available from the XXX database (accession number(s) XXX, XXX.*).<br>• If the data are all contained **within the manuscript and/or Supporting Information files**, enter the following: *All relevant data are within the manuscript and its Supporting Information files.*<br>• If neither of these applies but you are able to provide **details of access elsewhere**, with or without limitations, please do so. For example:<br><br>*Data cannot be shared publicly because of [XXX]. Data are available from the XXX Institutional Data Access / Ethics Committee (contact via XXX) for researchers who meet the criteria for access to confidential data.*<br><br>*The data underlying the results presented in the study are available from (include the name of the third party*	All relevant data are within the manuscript and its Supporting Information files.

and contact information or URL).<br>• This text is appropriate if the data are owned by a third party and authors do not have permission to share the data.<br><br>* typeset	
Additional data availability information:	

Powered by Editorial Manager® and ProduXion Manager® from Aries Systems Corporation

Manuscript

Click here to access/download;Manuscript;MS_Phenotypic characterization_PLOS.doc

Title: Characterization of Indigenous Chicken Phenotypes in Liban Jawi District, Ethiopia: *A Qualitative and Quantitative Analysis*

By

[1]Desalegn Begna, Policy Studies Institute, Ethiopia, E-mail: dbegna67@gmail.com

[2]Teferi Bacha, Ambo University, Mamo Mezemir Guder Campus, Ethiopia, Email-tefaribacha@gmail.com

[2]Shamble Boki, Ambo University, Mamo Mezemir Guder Campus, Ethiopia, Email-dshambelboki@yahoo.com

[1]Corresponding author: Desalegn Begna: E-mail: dbegna67@gmail.com

Abstract

Indigenous chickens play a crucial role in the livelihoods of smallholder farmers in rural Ethiopia. This study aimed to phenotypically characterize indigenous chickens in the Liban Jawi district, focusing on phenotypic characteristics measurements. A total of 224 chickens were selected from a larger population of 2452 chickens in the study areas. The selected chickens were segregated by sex and agroecologies, and comprehensive phenotypic characteristics measurements were taken.

The analysis of qualitative traits revealed that the male chickens exhibited a plain head shape (54.7%), single combs (39.5%), red feather plumage (26.7%), yellow shanks (54.7%), red earlobes (51.6%), and white skin (68.6%). Among the female chickens, single combs (75.4%), red earlobes (51.4%), yellow shanks (50.1%), and brown mottled feathers (23.9%), with red (23.1%) and black (9.4%) feathers observed.

The average body weight of cocks was 1.46±0.02 kg, while hens were 1.21±0.01 kg. Cocks exhibited larger body dimensions, with average body lengths, body widths, shank lengths, shank circumferences, and wingspans measuring 38.05±0.26 cm, 22.20±0.20 cm, 8.76±0.11 cm, 3.58±0.05 cm, and 38.56±0.13 cm, respectively. Correspondingly, hens had an average of 31.55±0.33 cm in body length, 19.04±0.21 cm in body width, 6.66±0.05 cm in shank length, 3.36±0.03 cm in shank circumference, and 32.98±0.13 cm in wingspan. Particularly, shank length emerged as the best predictor of body weight for both hens (r=0.45) and cocks (r=0.44).

The study revealed significant variations in both qualitative and quantitative traits among the indigenous chicken population, suggesting the potential for improvement through selective breeding at the community level. These findings provide valuable insights into the phenotypic characterization of indigenous chickens in the study district, serving as a foundation for future breeding programs and conservation efforts aimed at enhancing productivity and preserving genetic diversity. Further molecular-level characterization of the indigenous chicken population is recommended to validate the current phenotypic results.

Keywords: Quantitative, Indigenous, Chicken, Qualitative, Phenotypic

1. **Introduction**

Indigenous chicken (Gallus domesticus) constitutes approximately 80% of the global poultry population, with the majority found in developing countries (Melesse, 2014). In Africa, village fowl, including indigenous chickens, make up more than 85% of the total fowl population, with each village household typically keeping between 5 and 20 birds (Tadelle, 2003). Ethiopia, in particular, has a significant chicken population, estimated at around 56.99 million, with approximately 78.85% being indigenous breeds (CSA, 2021). The high proportion of indigenous chickens highlights their importance as a genetic resource in the country. Within Ethiopia, the West Shewa zone, including the Liban Jawi district, contributes about 1.3 million chickens to the total population of 16.7 million in Oromia (CSA, 2021).

Under rural chicken production systems, the average flock size ranges from 7 to 10 birds per household, consisting of adult hens, a cock, and various age groups of growers (Melesse & Negesse, 2011). Indigenous chicken populations in Ethiopia have been characterized based on various criteria, such as plumage color and geographic region of sampling (Teketel F, 1986; Tadelle, 2003; Halima et al., 2007). Understanding the unique traits, adaptation to the environment, and socio-cultural importance of indigenous chickens is essential for their conservation and utilization (Melesse & Negesse, 2011).

Phenotypic variations in major morphological traits, including plumage color, feather contours, shank and ear-lobe colors, and comb types, are common among indigenous chicken populations (FAO, 2012). However, these native chickens generally exhibit poor egg production performance, delayed maturity, and extended broodiness, as they have primarily been selected for their adaptive traits (Moges et al., 2010). In Ethiopia, there has been limited research on the characterization and classification of indigenous non-descriptive chicken ecotypes, and efforts to identify and evaluate these genetic resources are still in their early stages (FAO, 2012).

While there are few examples of breeding programs for indigenous chicken breeds in Africa, recent initiatives have focused on genetic improvement through selective breeding in Ethiopia to enhance the productivity of indigenous chickens and conserve genetic diversity (FAO, 2012). Phenotypic characterization provides valuable data on the current and potential future uses of indigenous chickens in a given area. Therefore, the objective of this study is to conduct a phenotypic characterization of indigenous chickens in the Liban Jawi district, focusing on qualitative traits, body weight, and various linear body measurements.

2. Methodologies

The study was conducted in the Liban Jawi district in the West Shewa zone of the Oromia Regional State, Ethiopia (Figure 1). The district was purposively selected based on its potential for the indigenous chicken population and the rapid distribution of crossbred and exotic chicken breeds, which could impact indigenous chickens' population size and adaptive traits. The district is approximately 171 km west of Addis Ababa and 51 km from the zone administration in Ambo town. It shares borders with the Mida Kegni, Jibat, Toke Kutaye, and Chaliya districts. The district covers an area of about 311.7 km², with land use distribution consisting of approximately 57% cultivation, 17% grazing, 26% forests, and 1% allocated for other purposes. The district experiences a mean annual temperature ranging from 10°C to 25°C, and annual rainfall ranges from 900 to 1800 mm. The altitude in the area varies between 1100 and 2900 meters above sea level (m.a.s.l). The district was categorized into three agroecological zones based on altitude and temperature: highland (44%), midland (31%), and lowland (25%), consisting of eight, five, and three kebeles, respectively. Roge Danisa and Roge Ajo from the highland, Liban Gamo from the midland, and Haro Marami from the lowland areas, in total four kebeles were purposively selected based on their potential for indigenous chicken production.

Figure 1*:* Map of the study areas

Data collection involved field visits to the district and target sampling kebeles, where indigenous chickens were observed and measured. The sample population was selected using purposive sampling.

To determine the sample size, the number of households with at least two matured indigenous chickens (hens and/or cocks) was listed. Thus, a total of 2166 households and 2452 chickens were surveyed in the four kebeles and from this population, a sample of 224 chickens was selected from 192 households having at least two matured checkin. The sample size for the study was determined according to Arsham's (2002) formula, which is given by $N = \frac{0.25}{SE^2}$, where N represents the sample size and SE denotes the standard error of the sample size. In this study, a standard error of 0.036 was used with a 95% confidence level, resulting in a sample size of 192 households. Following the phenotypic characterization guidelines provided by the FAO (2012), it was recommended to include 100-300 matured females and 10-30 matured males to maintain validity. Therefore, a total of 224 (56 from each kebele) matured indigenous chickens were sampled, consisting of 138 females and 86 males. Both qualitative and quantitative measurements were taken from these sampled chickens.

Qualitative traits, which are observable characteristics that do not involve numerical measurement, were collected through visual observation of traits such as head shape,

comb type, feather plumage, shank color, earlobe color, and skin color. Whereas, quantitative measurements were taken using a flexible tape meter for body weight and various linear body dimensions, including body length, body width, shank length, shank circumference, and wingspan.

The collected data was organized, cleaned, and processed to ensure accuracy and usability and analyzed using the Statistical Package for Social Sciences (SPSS Ver.20) considering variables such as sex and agroecology as independent variables. These software packages offer a range of statistical techniques and tools to explore relationships, test hypotheses, and generate results.

$Yij= \mu + Ai + Bj + ABij + eij$ Where

Yijk: the corresponding quantitative trait of local chicken in ith agro-ecology (i=3, highland, midland & lowland) of jth sex (j= 2, male and female)

μ: overall population mean for the corresponding quantitative trait

Ai: effect of ith agro-ecology

Bj: effect of jth sex (j=2, male & female)

ABij: agro-ecology & sex interaction effect

eij: residual error (Kosgey, I .S. 2004)

Descriptive statistics were generated to provide insights into the phenotypic characteristics of the indigenous chicken population in the study area.

3. RESULTS AND DISCUSSION

3.1. General Socio-Economic Characteristics:

The socio-economic characteristics provide insights into the demographic composition of the respondents and their roles in chicken management within the different agroecologies of the study area. The socio-economic characteristics across the three agroecologies of this study are presented in Table 1. Generally, the socioeconomic characteristics highlighted the limited education among respondents, the lack of awareness of proper chicken management, and the prevalence of Protestantism in the study area. The majority of the respondents in all three agroecologies were females (68.8%) and this finding is consistent with Meseret (2010) that reported 70% are female poultry keepers and this suggests that females are the primary role players compared to males.

Of all the respondents, around 26.6% and about 11% fall within the age groups of 31-40 and 51-65 years, respectively, highlighting the presence of a productive age group for effective chicken management. Age affects the level of experience, knowledge, and ability to adapt to new farming practices(Gauchan et al., 2020).

The educational background of the interviewed respondents varied, with approximately 50% having limited education, including the ability to read and write and attending

religious schools. Another 12% did not have any formal schooling. Around 24% of respondents had attended primary school (grades 1-8), while 14% had completed high school (grades 9-10). Education plays a crucial role in improving the overall performance, sustainability, and profitability of the chicken production business (Attia et al., 2022; Ebsa et al., 2019). The educational level plays an important role in accessing information, adopting modern techniques, and making informed decisions related to poultry management, disease control, and market trends. Higher levels of education are often associated with better farm management practices and increased productivity (Gauchan et al., 2020).

The majority of respondents (88.6%) were engaged in mixed crop-livestock production systems and kept chickens for immediate income generation to cover various expenses, such as purchasing salt, coffee, clothes, and medication. Similar findings have been reported in other parts of Ethiopia (Dessie & Ogle, 2001; Sewunet, 2015; Wondmeneh, 2015).

In terms of religion, 57.3% of the surveyed population identified as Protestant, while 31.7% were Orthodox and 10.9% were Waqefataa, indicating more Protestant habituating in the study area and religion could influence poultry meat consumption (Seleshe et al., 2014).

The family size per household did not show any significant differences ($p < 0.05$) between the highland and midland agroecologies with an overall mean of 4.65±0.18, it is established that household size impacts the available labor force and resources for investment in poultry production and influences the income earned from poultry farming (Gauchan et al., 2020).

Table 1: Respondents and owners' profile in the study area

Respondent profile	Agroecological zone High altitude n=96	Mid altitude n=48	Low altitude n=48	Total n =192
Sex				
Male	24 (25)	20 (41.7)	16 (33.3)	60 (31.2)
Female	72 (75)	28 (39.3)	32 (56.7)	132 (68.8)
**Age of respondent**				
<20	18 (18.8)	11 (22.9)	11 (22.9)	40 (20.8)
20-30	18 (18.8)	12 (25)	12 (25)	42 (21.9)
31-40	30 (31.2)	10 (20.8)	11 (22.9)	51 (26.6)
41-50	19 (19.7)	13 (27.1)	6 (12.5)	38 (19.8)
51-65	11(11.5)	2 (4.2)	8 (16.7)	21 (10.9)
Educational level of respondents				
Unschooled	12 (11.5)	5 (10.4)	6 (12.5)	23 (12)
Religious school	14 (14.6)	11 (22.9)	3 (6.2)	28 (14.6)
Writing and reading	34 (35.5)	16 (33.3)	20 (41.7)	70 (35.4)
Primary(1-8)	24 (25)	10 (20.8)	11 (22.9)	45 (24)
High school	12 (12.5)	6 (12.6)	8 (16.7)	26 (14)
**Farming systems**				
Crop production	3(3.2)	5(10.4)	3(6.3)	11(5.7)
Livestock production	7(7.3)	2(4.2)	2(4.2)	11(5.7)
Mixed farming systems	86(89.5)	41(85.4)	43(89.5)	170(88.6)
**Religious of respondents**				
Protestant	55(57.3)	31(64.6)	24(60.4)	110(57.4)
Orthodox	31(32.3)	13(27.1)	17(35.4	61(31.7)
Waqeffataa	10(10.4)	4(8.3)	2(4.4)	21(10.9)
Family size/HH (Mean ± S E)	4.52±0.16	4.81 ±0.17	5.25±0.21	4.65±0.18 ns
Landsize/HH (ha.) (Mean ± SE)	1.54±0.12	1.73±0.19	2.34±0.22	1.87±0.17 **

n= Number of respondents **=significance, number in the parentheses show % of respondent.

The study found that the mean family size in the study area was 4.65±0.18, which is similar to the average of 5.5±0.22 reported for the Oromia region (CSA, 2019). The average land size per household was 1.87±0.17 hectares, indicating sufficient land for agricultural activities. Notably, lowland areas had the largest average land size per household (2.34±0.22ha), while highland areas had the smallest (1.54±0.12ha), with a significant difference ($p<0.05$). However, there was no significant difference ($p > 0.05$)

between midland and the other two agroecologies. The land holding size in the area was considerably larger than the 0.86 ha reported in the Dale, Wonsho, and Loka Abaya districts of South Ethiopia (Mekonnen, 2007), but consistent with the national average of 1.18 ha (CSA, 2019), and correlated closely with population density (Gashe Desta, 2023; Yigezu Wendimu, 2021). Highland and midland agroecologies were found to be more suitable for both crop and animal production, while lowland areas faced challenges due to limited animal feed caused by low rainfall (Gashe Desta, 2023).

3.2. Phenotypic characterization

3.2.1. The qualitative trait of indigenous chickens

The current study found that the indigenous chickens in the study area were predominantly normally feathered, with 100% of females and 100% of males exhibiting this trait (Table 2). This finding is consistent with the previous research conducted by Nigussie Dana (2011), who also observed that most indigenous chicken ecotypes were normally feathered. Additionally, a majority of the indigenous chickens in the study area had no feathered shanks, with only 33.7% of hens and 49.3% of cocks displaying feathered shanks. This observation aligns with the findings of Halima (2007), Bogale (2008), and Faruque et al. (2010), who reported that most indigenous chicken ecotypes lack shank feathers and have yellowish-colored shanks.

Table 2 and Figure 2 illustrate the diverse plumage coloration observed in the chicken population. The results indicate that the dominant colors for hens/females in the study area were brown mottled (23.9%), red (23.1%), black (9.4%), dark brown (7.9%), black laced white (6.5%), reddish brown (5.2%), and greyish mixture (5.1%). These findings differ from the reports of Halima (2007), who documented white, black, red, and black with white stripes as the prevalent plumage colors in North West Ethiopia. The wide variation in plumage coloration could be attributed to a lack of selection for specific traits in the local chicken population, a phenomenon also observed in Nigeria (Daikwo et al., 2011), Jordan (Abdelqader et al., 2007), and Botswana (Badubi et al., 2006). For cocks/males, the dominant plumage colors were red (26.7%), brown mottled (19.8%), dark brown (9.3%), black laced white (8.1%), brown mottled (5.8%), reddish brown (5.6%), and greyish mixture (4.7%). Duguma (2006) also reported a wide range of plumage coloration in indigenous chicken populations in Ethiopia and concluded that this diversity is a characteristic of adaptability and survival. Similarly, Odubote (2014) observed a similar pattern in Nigerian native chickens, highlighting the adaptability and survival benefits of diverse plumage colors, which can be attributed to the absence of targeted selection breeding programs for specific colors.

Red was the predominant plumage color in male chickens, accounting for approximately 25.1% in highland, 33.3% in midland, and 23.8% in lowland agroecologies. This finding is consistent with the report by Halima Hassen (2007), who highlighted that red was the dominant plumage color in indigenous chickens, comprising 29.00% of the population.

Black, white, and yellowish-brown, were also present but in lower proportions. A notable characteristic in males was the presence of a black breast, which was almost absent in females.

In terms of comb types, single comb was the predominant type in males, accounting for 39.5%, followed by pea comb at 37.2%. Among females, 75.4% had single combs, with pea combs being the second most common at 22.5%. These results align with the findings of Tadele et al. (2018) in the Caffa zone of southwestern Ethiopia, where single comb was the predominant type in both males (59.7%) and females (72.2%) (Figure 2).

Figure 2: Variations of comb type among indigenous chickens

There were significant differences in head shape between male and female chickens ($p<0.01$) and among the studied agroecologies ($p<0.05$). Approximately 54.7% of males and 59.4% of females in the study agroecologies had a plain head shape. However, there was no significant difference ($p > 0.05$) in plain head shape among the agroecologies. This finding differs from the report by Nigussie (2010), who observed that crest heads were predominant in the northern population (75% in Farta) and plain heads were characteristic of the southern population (64% in Konso and 71% in Sheka). This discrepancy may be attributed to breed differences.

In males, the predominant shank color was yellow, accounting for 54.5% of highland, 47.6% of midland, and 61.9% of lowland agroecologies. Similarly, in female chickens, yellow shank color was dominant, with proportions of 50.7% in highland, 54.3% in midland, and 44.4% in lowland agroecologies (Table 2). The observed shank colors showed significant differences ($p < 0.05$) among the agroecologies for both males and females. These findings align with the reports of Aberra Mellese and Tegene Negesse (2011) that highlighted yellow shank color as the dominant trait in southern regions of Ethiopia, as well as the observations of Halima (2007) regarding the prevalence of yellow shank color in Ethiopian indigenous chickens.

In terms of earlobe color, both males (51.6%) and females (51.4%) with red earlobes were dominant in the studied agroecologies. Yellow and brown earlobe colors were observed but in minimal proportions across all three agroecologies (Table 2). There were highly significant differences ($p < 0.03$) in earlobe color among the chickens in the agroecologies for both males and females (Figure 4). These results disagree with the observations of Duguma (2006) and Egahi et al. (2010) from the western and eastern parts of Ethiopia and Nigeria, respectively.

Figure 3: White ear lobe and brown plumage color hen (A), and white ear lobe and black-laced white plumage color hen/female/ (B)

There was inconsistency in plumage color and ear lobe between the chickens in the study areas. As shown in Figure 4, there is brown ear lobe, black plumage color, pea combed type, and red eye color (A) and white plumage color, red ear lobe, and eye with single comp type (B) and this finding of multiple variants of plumage colours agrees with Dana et al., (2010).

Figure 4: Shows inconsistency in plumage color and ear lobe

(A), Brown ear lobe, black plumage color, pea combed type, and red eye color, (B), white plumage color, red ear lobe, and eye with single comp type)

Significant differences ($p < 0.003$) in skin color were observed across the agroecologies for both male and female chickens. White was the predominant skin color in males (70.5%, 68.1%, 76.2%) and females (54.3%, 57.1%, 47.1%) in highland, midland, and lowland agroecologies, respectively. Yellow skin color was rarely observed in both males and females across all three agroecologies (Table 2).

Table 2: Qualitative traits of indigenous chicken in the study area

	Agro ecology								
Qualitative trait	Highland		Midland		Lowland		Overall		*p*-value
	Cock%	Hen=%	Cock=%	Hen=%	Cock=%	Hen=%	Cock	Hen %	
Plumage Color									0.009
Black	6.8	10.1	4.8	5.7	4.8	4.2	5.8	9.4	
Black mottled	6.8	4.3	No	No	No	5.7	3.5	3.6	
Black-laced white	6.6	4.3	No	8.6	19.1	8.6	8.1	6.5	
Brown	9.1	1.4	No	5.7	3.5	5.7	5.8	3.6	
Brown mottled	18.2	24.6	14.3	28.1	28.6	14.3	19.8	23.9	
Dark brown	6.7	8.7	19	2.9	2.3	11.4	9.3	7.9	
Dark Brown mottled	7.1	4.3	9.5	2.1	No	2.9	5.8	3.6	
Greyish mixture	0	5.8	17	2.7	No	5.7	4.7	5.1	
Red	25.1	15.9	31.1	31.4	23.8	28.6	26.7	23.1	
Reddish brown	4.5	10.1	No	1.4	14.3	No	5.6	5.2	
Wheaten	0	1.4	No	No	1.3	No	No	3.6	
Wheaten mottled	2.3	No	No	4.2	No	1.2	1.4	1	
White	2.3	5.8	4.3	4.1	2.3	6	1.2	1.2	
White mottled	4.5	3.3	No	3.1	No	5.7	2.3	2.3	
Comb Type									0.608
Single	34.1	72.5	38.1	74.3	52.4	82.4	39.5	75.4	
Pea	38.6	24.6	38.1	22.9	33.3	17.6	37.2	22.5	
Rose	13.6	2.9	14.3	2.8	No	No	10.5	2.1	
Double	13.7	No	9.5	No	14.3	No	12.8	No	
Head Type									0.017
Crest	47.7	44.9	47.6	40.0	38.1	32.4	45.3	40.6	
Plains	52.3	55.1	52.4	60.0	61.9	67.6	54.7	59.4	
Ear lobe Color									0.037
Red	54.5	53.6	38.1	48.6	57.1	50	51.6	51.4	
White	43.2	31.9	33.3	28.6	42.9	32.4	40.7	31.2	

Yellow	2.3	2.9	14.3	No	No	8.8	4.7	3.6	
Brown	No	11.6	14.3	22.8	No	8.8	3	13.8	
Eye Color									0.007
Pearl	No	7.2	14.3	11.4	No	2.9	3.5	7.2	
Brown	25	4.4	33.3	No	33.3	38.2	29.1	11.6	
Orange	29.5	40.6	28.6	42.9	23.8	14.7	27.9	34.8	
Red	45.5	47.8	23.8	45.7	42.9	44.2	39.5	46.4	
Shank Color									0.028
Black	20.5	22.4	23.8	31	14.3	25.2	19.8	36.2	
White	15.9	26.8	19	16	23.8	26.5	18.6	23.6	
Grey	9.1	No	9.6	2.9	No	4.2	6.9	0.2	
Yellow	54.5	50.8	47.6	50.1	61.9	44.1	54.7	40	
Skin Color									0.003
White	70.5	68.1	76.2	54.3	57.1	47.1	68.3	59.4	
Yellow	28.2	26.1	23.8	40	40.9	52.9	30.4	35.5	
Pink	1.3	5.8	No	5.7	2	No	1.3	5.1	
Feather Distribution									
Normal	100	100	100	100	100	100	100		
Shank feather									0.06
Present	27.3	40.6	33.3	57.1	47.6	58.8	33.7	49.3	
Absent	72.7	59.4	66.7	42.9	52.4	41.2	66.3	50.7	

No = not observed

3.2.2. Quantitative traits

The body weight and linear body measurements of chickens in the study area are summarized in Table 3. The mean body weight values for males and females were found to be 1.46±0.027 kg and 1.12±0.012 kg, respectively. These results showed significant differences ($p<0.05$) across all agroecologies for both sexes. The findings of this study are consistent with previous reports by Fayeye et al. (2006), Dana et al. (2010), Ibrahim (2011), and Andualem et al. (2020), which also observed similar mean body weights for chickens Ethiopia. However, it differs from the observations of Abebe Hailu et al. (2017) in the Guji Zone of Ethiopia, who reported higher body weights of 2.1±0.05 kg for males and 1.5±0.02 kg for females. These variations in body weight may be attributed to differences in the genetic background of local chicken populations, management practices, and the availability of feed resources in different regions (Tadelle et al., 2000).

Sexual dimorphism was observed in average body weight, with males (cocks) weighing higher than hens, which is consistent with the findings reported by Halima et al. (2007) and Dana et al. (2010). Body weight is an economically important trait for livestock farmers, and there is increasing interest in selecting for higher body weight to improve productivity in indigenous chicken populations (Peters et al., 2007).

The average body width and body length of indigenous chickens were measured to be 22.20±0.20 cm and 19.04±0.21 cm, respectively, in males, and 38.05±0.269 cm and 31.55±0.33 cm, respectively, in females. These measurements showed significant differences ($p<0.05$) across all agroecologies for both sexes. The average shank length in males was 8.76±0.110 cm, which was slightly lower than the values reported by Halima et al. (2007) in Northwestern Ethiopia (8.91 cm) and by Melesse & Negesse (2011) in Southern Ethiopia (9.1 cm). The body weight to shank length ratio is an important indicator of the degree of fleshing relative to body size, with higher ratios indicating heavier birds (Renema et al., 2007).

In the current study, the shank circumference values were measured to be 3.58±0.05 cm in males and 3.36±0.03 cm in females. These values are consistent with those reported for Wareng-Tangerang Indonesian local chickens (Mulyono, Sartika, and Nugraha, 2009), but higher than the values reported by Halima et al. (2007) in Northwestern Ethiopia. Sexual dimorphism in various morphological traits has been observed in local chicken populations, as reported in the literature by Msoffe et al. (2004), Fayeye et al. (2006), Halima et al. (2007), Dana et al. (2010), and Youssao et al. (2010).

The wing span of the indigenous chickens raised in the study agroecologies did not show significant differences ($p > 0.05$).

Table 3: Linear body measurements of male and female chicken populations (N=224)

Morphometric traits	Sex	Agroecologies					
		Highland nm= 44 nf = 68	Midland nm= 44 nf = 68	Lowland nm= 44 nf = 68	Overall nm= 138 nf = 86	F-v	P-values
Bodyweight(kg)	M	1.54±0.04	1.43±0.04	1.33±0.02	1.46±0.02	5.449	0.006
	F	1.15±0.017	1.21±0.02	1.22±0.02	1.21±0.01**	2.909	0.05
Body lengths(cm)	M	37.38±0.48	38.49±0.23	39.0±0.18	38.05±0.26	3.705	0.029
	F	32.65±0.42	31.11±0.68	29.85±0.68	31.55±0.33**	6.502	0.002
Body width(cm)	M	21.88±0.22	21.90±0.29	23.14±0.61	22.20±0.20	3.566	0.033
	F	18.78±0.30	19.68±0.47	18.88±0.34	19.04±0.21 Ns	1.609	0.204
Shank length(cm)	M	8.53±0.16	8.72±0.23	9.31±0.17	8.76±0.11	4.368	0.016
	F	6.6±0.08	6.81±0.11	6.64±0.11	6.66±0.05*	1.211	0.301
Shank. Circ	M	3.57±0.08	3.66±0.104	3.52±0.09	3.58±0.05	.39	0.672
	F	3.37±0.06	3.31±0.06	3.37±0.06	3.36±0.03 Ns	.273	0.762
Wingspan(cm)	M	38.65±0.21	38.51±0.25	38.43±0.23	38.56±0.13	.223	0.801
	F	33.09±0.18	33.06±0.26	32.69±0.27	32.98±0.13 Ns	.809	0.448
Comp length(cm)	M	2.20±0 .04	2.55±0.08	2.36±0.07	2.32±0.040	7.665	0.001
	F	2.02±0.04	2.15±0.02	2.15±0.27	2.08±0.024**	3.930	0.022
Beak length(cm)	M	1.85±0.03	1.96±0.04	1.99±0.03	1.91±0.023	3.660	0.030
	F	1.31±0.02	1.25±0.032	1.23±0.03	1.27±0.019 Ns	1.527	0.221

M=male, F=female, nm = number of male, nf= number of female, F-V= F-values, ns=non significance, *significance (*p*<0.05) and ** significance at (*p*<0.01).

3.2.3. Correlations of Body Weight and Linear Body Measurements

In the present study, positive correlations were observed between all body measurements for both sexes, as shown in Table 4. These correlations align with the findings of Apuno et al. (2011). Specifically, a high correlation was observed between body weight and shank length (r = 0.45), which is consistent with the report by Nagassa et al. (2014) on other indigenous chicken populations in Ethiopia. Additionally, positive and significant correlations were found between body weight and body length, body width, and wing span (r = 0.44, 0.43, 0.41, $p < 0.01$), respectively.

In animal breeding, it is common to predict body weight from linear measurements (Ogah et al., 2009). Therefore, it can be inferred that selection for any of these linear body parameters would directly contribute to improvements in body weight. Peters et al. (2007) emphasized that body weight is an economically important trait for livestock farmers, which is why there is an increasing focus on selecting higher body weight to improve productivity in indigenous chicken populations.

Table 4: Pearson Correlations of Body Weight and Linear Body Measurements of the study area

Variable	BW	BL	BD	SHL	SHC	WS	CL	BKL
BW	1	.44**	.425**	.452**	.309**	.416**	.153	.279**
BL		1	.649**	.674**	.453**	.721**	.315**	.537**
BD			1	.697**	.290**	.617**	.235**	.443**
SHL				1	.231**	.753**	.320**	.646**
SHC					1	.429**	.197**	.228**
WS						1	.337**	.720**
CL							1	.300**
BKL								1

**. Correlation is significant at the 0.01 level (2-tailed). *. Correlation is significant at the 0.05 level (2-tailed). BW= Body Weight, BL= Body Length, BD=Body width; WS= Wing Span, CL= comp length ShL= Shank Length, ShC=Shank Circumference, BKL=beak length

4. Conclusions and Recommendations

4.1. Conclusions

The phenotypic characterization of indigenous chickens in the Liban Jawi District, Ethiopia revealed several important findings.

In this study, sexual dimorphism was observed, with male chickens (cocks) weighing more than female chickens (hens).

There were positive correlations found between body length, body width, shank length, and wing span measurements, indicating that changes in one parameter are likely to be reflected in others.

There are significant positive correlations between body weight and other linear body measurements, suggesting that selecting for any of these parameters could lead to improvements in body weight.

These findings emphasize the significance of considering both qualitative (sexual dimorphism) and quantitative (body weight and linear measurements) aspects of chicken phenotypes. This knowledge is crucial for developing effective breeding strategies and enhancing productivity in the local poultry industry. By recognizing and utilizing these phenotypic traits, farmers and breeders can make informed decisions to improve the genetic potential of indigenous chickens, resulting in increased yields and sustainable livelihoods.

4.2. Recommendations

Based on the study of the phenotypic characterization of indigenous chickens in Liban Jawi District, Ethiopia, the following recommendations can be made:

Implement a selective breeding program that focuses on improving body weight in indigenous chicken populations.

Recognize and account for sexual dimorphism in indigenous chickens. Since male chickens tend to have higher body weights than females, it may be beneficial to separate them during rearing and allocate resources accordingly.

Educate local farmers on the importance of phenotypic characterization and selection for desired traits.

Preserve and conserve the genetic diversity of indigenous chicken populations in the region to ensure their resilience and sustainability in the face of changing circumstances, such as climate change or disease outbreaks.

Conduct additional research to explore the underlying genetic factors contributing to phenotypic variations in indigenous chickens.

Data Availability: The data supporting the findings of this study are primarily included in the article. Additional data that may not be included can be made available by contacting the corresponding author upon request.

Funding source: The research did not receive specific funding but was self-sponsored and performed as part of the MSc thesis of the authors.

Conflicts of Interest: The authors declare that they have no conflicts of interest.

Acknowledgments: The authors would like to extend their appreciation to Mamo Mezemir Campus of Ambo University, Ethiopia for permitting to conduct the case as MSc study.

REFERENCES

1. Abdelqader Abdu, Wollny C.B.A. and Gauly M.2007.Characterization of local chicken production systems and their potential under different levels of management practice in Jordan.Tropical Animal Health and Production .2007; 39:155–164.
2. Abebe Hailu, Manaye Misganaw, Abrahm Assefa, Fassil.2017. "On-farm phenotypic characterization of indigenous chicken populations" in Guji zone of oromia national regional state, Ethiopia. International Journal of Development Research,7, (11), 16652-16661.
3. Aberra M. and Tegene N. (2007): Study on the characterization of local chicken in Southern Ethiopia. Proceedings held in Awassa, 16-17, March 2007.
4. Andualem Yihun, Manzoor Ahmed Kirmani, Meseret Molla (2020). Phenotypic characterization of indigenous chicken Ecotypes in Awi Zone, Ethiopia, *Ecology and Evolutionary Biology*. Vol. 5, No. 4, pp. 131-139. doi: 10.11648/j.eeb.20200504.13
1. Apuno, A.A., Mbap, S.T. & Ibrahim, T. (2011).Characterization of Local Chickens (Gallus Gallus Domestics) in Shelleng and Song Local Government Areas of Adamawa State, Nigeria .Agriculture and Biology Journal of North America, 2(1):6-14.
5. Attia, Y. A., Rahman, M. T., Shehata, A. A., Hafez, H. M., Hossain, M. J., Basiouni, S., & Khafaga, A. F. (2022). Poultry Production and Sustainability in Developing Countries under the COVID-19 Crisis: Lessons Learned. *Animals*, *12*(5). https://doi.org/10.3390/ani12050644
2. Badubi S S, Rakereng M and Marumo M. (2006). Morphological characteristics and feed resources available for indigenous chickens in Botswana. Livestock Research for Rural Development, Volume 18
3. Bogale, K. (2008). In Situ Characterization of Local Chicken Eco-Type for Functional Traits and Production System in Fogera Woreda, Amhara Regional State.Msc.Thesis Submitted to Haramaya University, Haramaya, Ethiopia.
4. CSA. 2021. Agricultural sample survey 2019/20. Report on livestock and livestock Characteristics
5. CSA. 2019. Agricultural sample survey 2019/20. Report on livestock and livestock Characteristics, vol.2.statistical bulletin No. 468. Addis Ababa, Ethiopia.
6. Daikwo, I.S., Okpe, A.A. & Ocheja, J.O. 2011, Phenotypic characterization of local chickens in Dekina. Int. J. Poult. Sci., 10: 444–447
7. Dana, N., Dessie, T., van der Waaij, L. H., & van Arendonk, J. A. M. (2010). Morphological features of indigenous chicken populations of Ethiopia. *Animal Genetic*

Resources/Ressources Génétiques Animales/Recursos Genéticos Animales, *46*(September 2009), 11–23. https://doi.org/10.1017/s2078633610000652
8. Dana, N., Dessie, T., van der Waaij, L.H. & van Arendonk, J.A.M. 2010. Morphological features of indigenous chicken populations of Ethiopia. Anim. Genet. Resour. Inf., 46: 11–23
9. Dessie, T., & Ogle, B. (2001). Village Poultry Production Systems in the Central Highlands of Ethiopia. *Tropical Animal Health and Production*, *33*(6), 521–537. https://doi.org/10.1023/A:1012740832558
10. Duguma, R. (2006). phenotypic characterization of Some Indigenous Chicken Ecotypes of Ethiopia. Livestock Research for Rural Development. Vol. 18:131.
11. Duguma, R. (2009). Understanding the role of indigenous chickens during the long walk to food security in Ethiopia. Livestock Res.Rural Dev., 21(8). (available at http://www.lrrd.org/lrrd21/8/dugu21116.htm) Accessed 10 January 2013.
12. Ebsa, Y. A., Harpal, S., & Negia, G. G. (2019). Challenges and chicken production status of poultry producers in Bishoftu, Ethiopia. *Poultry Science*, *98*(11), 5452–5455. https://doi.org/10.3382/ps/pez343
13. Emebet, M., Singh, H., Sisaye, T., & Johansson, A. M. (2014). phenotypic characterization of Indigenous Chicken Population in South West and South Parts of Ethiopia. British Journal
14. Emebet, M.2(015). Phenotypic and Genetic Characterization of Indigenous Chicken in Southwest Showa and Gurage Zones of Ethiopia'.Ph.D. dissertation at Addis Ababa University pp.127.
15. FAO (2004). Manual small scale Poultry production technical guide by Sonaiya and Gunaratne E.B and Swan S.E.J.FAO, Animal Production and Health Series No.1.http://www.fao.org/docrep /008/y5169e/y 5169e00. HTM date accessed 04-09-2009.
16. FAO (2013). Poultry development. In *The role of poultry in human nutrition*.
17. FAO, (2012) Draft guidelines on phenotypic characterization of animal genetic resources Commission on Genetic Resources for Food and Agriculture.13th Regular Session, 1822, July 2011, Rome (availableathttp://www.fao.org/docrep/meetin, 651e.pdf)
18. Fayeye, T.R., Ayorinde, K.L., Ojo, V. & Adesina, O.M. (2006). Frequency and influence of some major genes on body weight and body size parameters of Nigerian local chickens. Livestock Res
19. Gauchan, D., Joshi, B. K., Sthapit, S., & Jarvis, D. (2020). The Journal of Agriculture and Environment. *The Journal of Agriculture and Environment*, *21*(June), 31–43. www.moald.gov.np
20. Halima H, Neser FWC. Kock A., Marle-Köster E., (2009). Study on the genetic diversity of native chickens in northwest Ethiopia using microsatellite markers. Afr. J. Biotech., 8: 1347-1353.
21. Halima Hassen (2007).Phenotypic and genetic characterization of indigenous chicken populations in North-West Ethiopia. Ph.D. Thesis, Submitted to the faculty of natural and

Agricultural Sciences Department of animal wildlife and grassland Sciences University of the Free State, Bloemfontein, South Africa. Pp 186

22. Mekonnen G/egziabher. (2007). Characterization of smallholder poultry production and marketing system of dale, Wonsho and Loka Abaya Weredas of southern Ethiopia. M.Sc Thesis, Awassa College of Agriculture, Hawassa University 95pp.
23. Melesse A. (2014). “Significance of scavenging chicken production in the rural community of Africa for enhanced food security”. *World’s Poultry Science Journal* 70.3, 593-606.
24. Melesse, A. & Negesse, T. (2011). Phenotypic and morphological characterization of indigenous chicken population in southern region of Ethiopia Anim.Genet. Resour. Inf., 49:19-31.
25. Meseret Molla (2010). Characterization of village chicken production and marketing system in Gomma wereda, jimma zone, Ethiopia, M.sc. thesis Meseret Molla Bogale jimma university, Ethiopia august, 2010jimma.PP
26. Nigussie D., (2013.)On-farm phenotypic characterization of indigenous chicken and chicken production systems in Southern Zone of Tigray, Northern Ethiopia, Thesis Submitted to the, School of Graduate Studies Haramaya University,Ethiopia,pp 106.of Poultry Sciences, 3 (1), 15-19.
27. Nigussie, D., (2011). Breeding programs for indigenous Production and Health, technical Livestock sector chicken in Ethiopia, Analysis of diversity in brief: Ethiopia. No. 1,
28. Seleshe, S., Jo, C., & Lee, M. (2014). Meat consumption culture in Ethiopia. *Korean Journal for Food Science of Animal Resources*, *34*(1), 7–13. https://doi.org/10.5851/kosfa.2014.34.1.7
29. Sewunet, Z. (2015). Institutions and policies to implement the Ethiopia livestock master plan. *Ethiopia LMP Brief*, *5*(August 2015), 1–4. https://cgspace.cgiar.org/bitstream/handle/10568/68038/LMP_5.pdf?sequence=1&isAllowed=y
30. Tadelle, D, T. Million, Alemu Yami and K.J. Peters, 2003.Village chicken production system in Ethiopia. University of Agricultural sciences, pp: 70 walk to food security in Ethiopia. Red, 19 (29.9), 21-5
31. Tadelle, D., Alemu, Y., & Peters, K. J. (2000). Indigenous chickens in Ethiopia: Genetic potential and attempts at improvement. *World’s Poultry Science Journal*, *56*(1), 52–54. https://doi.org/10.1079/wps20000005
32. Teketel F. (1986): Studies on the meat production potentials of some local strains of chickens in Ethiopia. Ph.D. Thesis, J. L. University Giessen, Germany, 123-126
33. Wondmeneh E.(2015): Genetic improvement in indigenous chicken of Ethiopia. Ph.D. Thesis, Wageningen University, NL (2015): 74

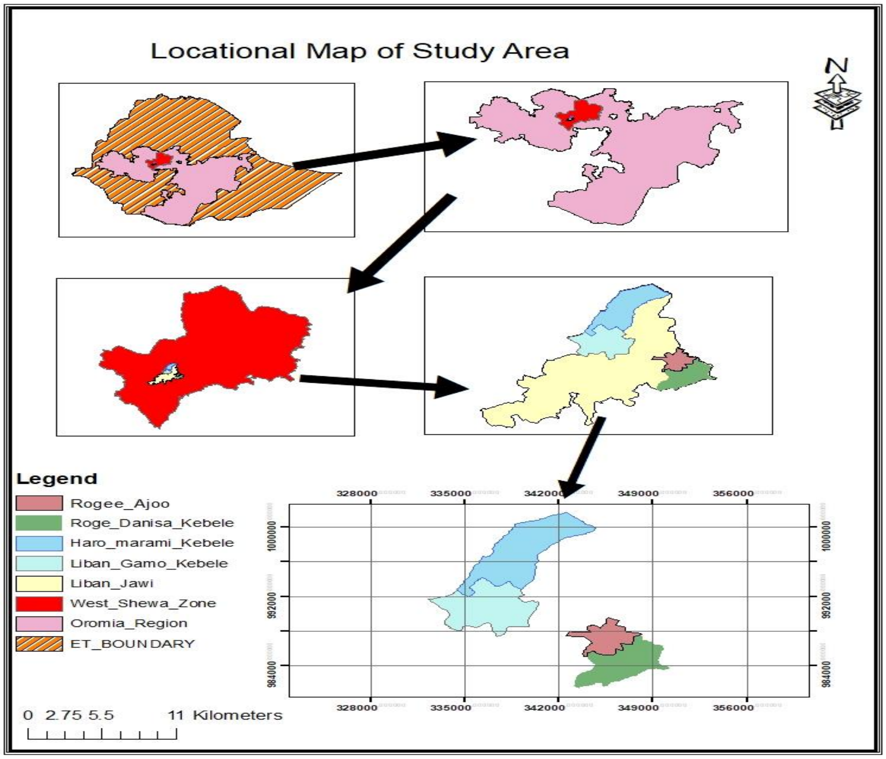

Figure 1: Map of the study areas

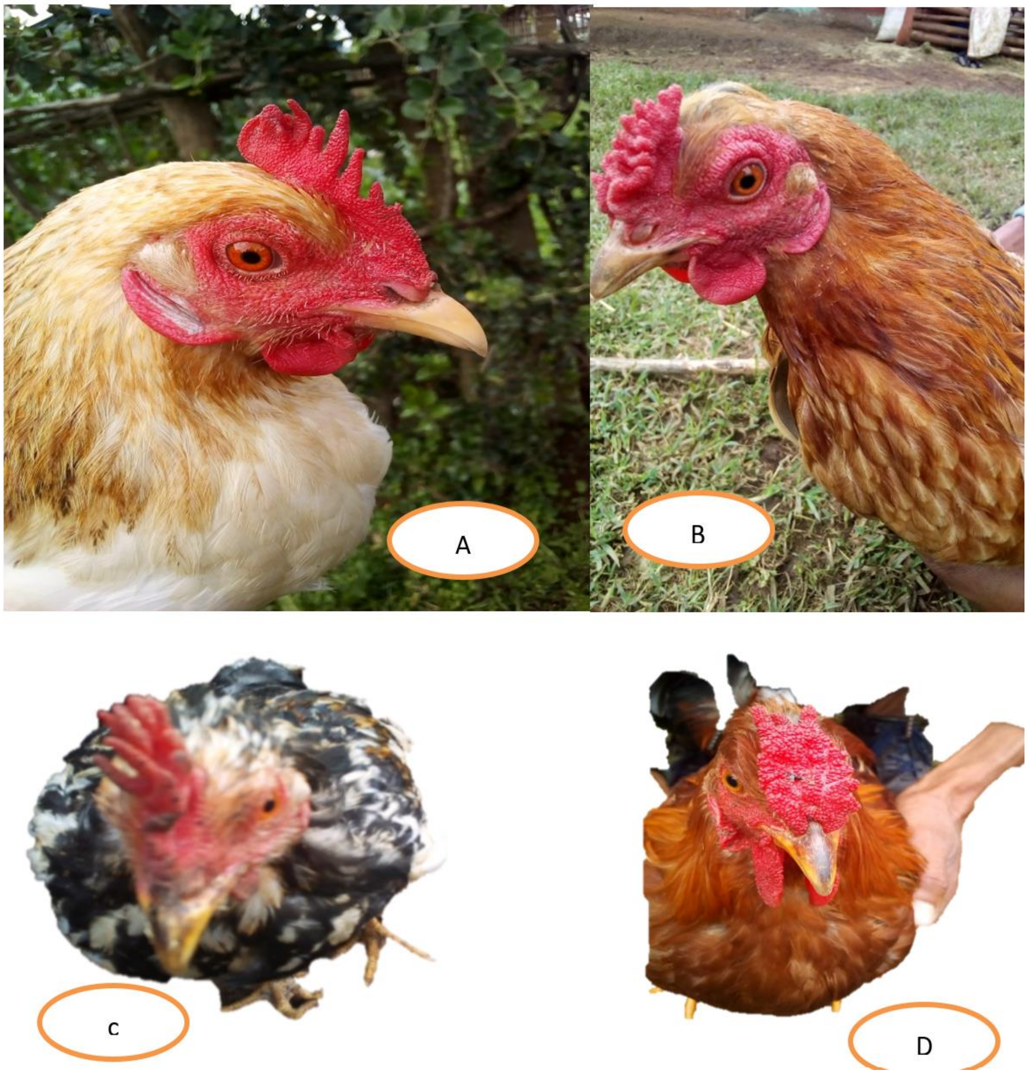

Figure 2: Variations of comb type among indigenous chickens

(A) Hen with a single comb, (B) hen with pea comb, (C) cock with a double comb (D) Cock with a rose comb

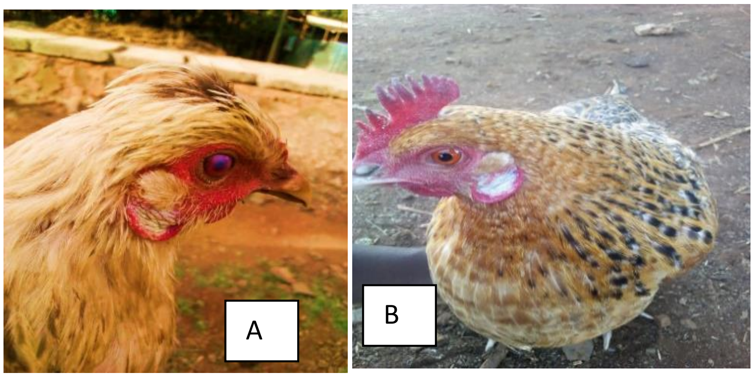

Figure 3: White ear lobe and brown plumage color hen (A), and white ear lobe and black-laced white plumage color hen/female/ (B)

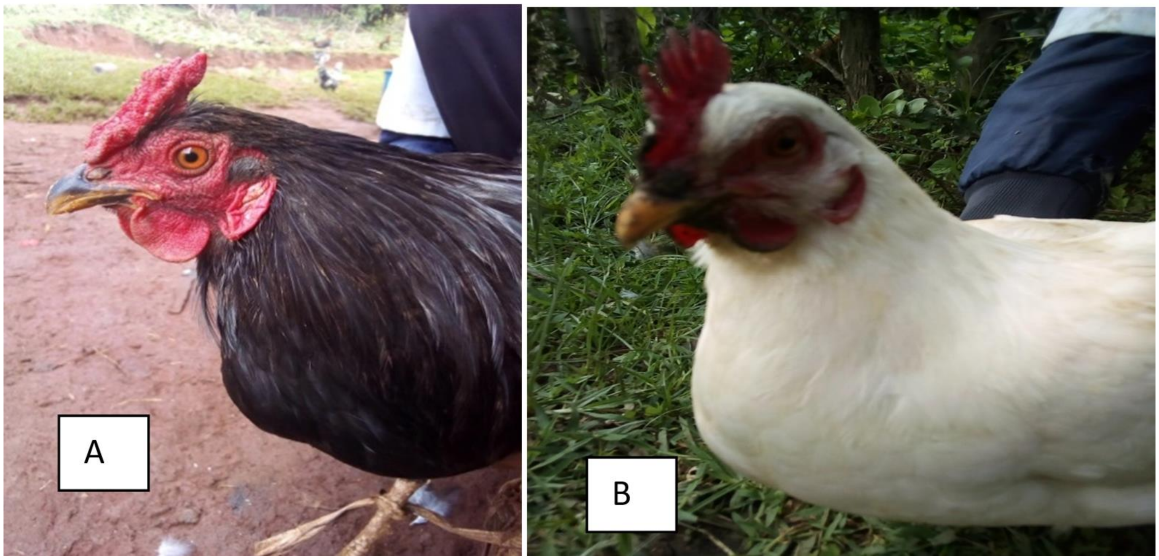

Figure 4: Shows inconsistency in plumage color and ear lobe
