# Supplementary material for: Characterization of indigenous chicken phenotypes in Liban Jawi District, Ethiopia: A qualitative and quantitative analysis
Source: PLoS One. 2025 Jan 10;20(1):e0307793. doi: 10.1371/journal.pone.0307793 (PMC11723647; doi:10.1371/journal.pone.0307793)
Supplement: Attachment — Submitted filename: General comments in Ms-word.docx [file pone.0307793.s003.docx]

Subject: Manuscript Refinement Suggestions for Submission to PLOS ONE

Dear Editor,

I have carefully reviewed the manuscript titled "Characterization of Indigenous Chicken Phenotypes in Liban Jawi District, Ethiopia: A Qualitative and Quantitative Analysis" submitted for consideration to PLOS ONE. The study offers valuable insights into the phenotypes of chickens, which could significantly contribute to the development of conservation and breeding programs. However, to enhance the quality and clarity of the manuscript, I recommend that the authors address the following points:

1. Clarification of the Problem Statement:

The authors are encouraged to provide a more detailed and clear elaboration of the statement of the problem under investigation.

2. Diversification of Citations:

It is advised that the authors include additional recent publications related to the phenotypic characterization of indigenous chickens in various regions of Ethiopia. To avoid redundancy, please refrain from citing similar references repeatedly.

3. Consistency in Referencing Style:

Ensure consistency in the referencing style throughout the manuscript.

4. Enhancements in Grammar, Spelling, and Formatting:

Please refine the grammar, spelling, and overall editing of the manuscript to align with the journal's guidelines. Additionally, ensure that tables and figures are correctly presented, with self-explanatory legends.

5. Detailed Methodology Section:

Separate the methodology section into sub-headings for data collection, sampling, and statistical analysis. Elaborate further on the statistical analysis procedures employed.

6. Comprehensive Discussion:

Clearly articulate the implications of the study in the discussion section.

7. Inclusion of Univariate and Multivariate Analyses:

It is recommended to incorporate more univariate and multivariate analyses to enrich the manuscript. Ensure that mean comparisons are conducted.

8. Consideration of Body Indices Calculation:

If feasible, include body indices calculations to provide insights into the overall conformation and functionality of the chickens studied.

9. Precision in Conclusions and Recommendations:

Ensure that the conclusions and recommendations align closely with the study results. Address the effects of agro-ecology, which should be adequately discussed throughout the manuscript, including in the abstract.

I believe that addressing these points will significantly improve the manuscript's quality and contribute to its potential acceptance for publication. Thank you for considering these suggestions.

Sincerely,
