# Supplementary material for: Characterization of indigenous chicken phenotypes in Liban Jawi District, Ethiopia: A qualitative and quantitative analysis
Source: PLoS One. 2025 Jan 10;20(1):e0307793. doi: 10.1371/journal.pone.0307793 (PMC11723647; doi:10.1371/journal.pone.0307793)
Supplement: Attachment — Submitted filename: Response to Editor Queries.docx [file pone.0307793.s004.docx]

**Response to Editor’s queries**

1. Data availability: The research data have been uploaded as a **supporting information file**
2. Regarding permission from the original copyright holder of Figure 1: **Removed and replaced by coordinates of the study area**
3. Regarding copyright permission on Figures 2, 3, and 4: **Separate letter written to the editor**
4. Concerning the comments on additional information regarding the permits obtained for the work in the methods section: **the section was revised as “The research was authorized by Ambo University as part of the MSc thesis project, with all necessary permissions secured in compliance with academic guidelines” and the statement included in the methodology section**
5. Figure 4: No more Figure 4  in the revised document
