# Supplementary material for: Characterization of indigenous chicken phenotypes in Liban Jawi District, Ethiopia: A qualitative and quantitative analysis
Source: PLoS One. 2025 Jan 10;20(1):e0307793. doi: 10.1371/journal.pone.0307793 (PMC11723647; doi:10.1371/journal.pone.0307793)
Supplement: Attachment — Submitted filename: review, chicken plos one.docx [file pone.0307793.s005.docx]

**Introduction:**

„According to (9))…” – please correct and give authors surnames beside citation number (9)

Also wrong style of citation in the same sentence :…and (10) reported…”

**Methodologies**

“Thus, a total of 2166 households and 2452 chickens were surveyed from which a total of 192 households and 224 chickens were selected” – why and how these households and chickens were selected?’

“Following the phenotypic characterization guidelines provided by (17)…” – the same situation as in introduction

Please explain what this formula is and why it was used. : Yij= μ + Ai +Bj +ABij +eij

Yij or Yijk?

“Descriptive statistics tests, such as chi-square and ANOVA…” – nor ANOVA, nor chi-square are not examples of descriptive statistics!

**Results and Discussion**

Please correct citation style in whole text (same mistake as in introduction and methodology)

“The chickens belonging to households headed by older farmers tended to exhibit more traditional plumage patterns and comb types, suggesting that age and traditional knowledge influence the conservation of certain cultural traits within the local chicken ecotypes and this is in line with (39), Similarly, the educational level of the household heads was positively correlated with the prevalence of improved production traits, indicating that more educated farmers are better able to implement management practices that support the expression of these economically valuable characteristics (40).” – The authors have made a socioeconomic analysis of the study area, but other than the characteristics of the area, there is no information on whether and how this would translate into improvements of local chickens. They only cite the literature. Do their results support other authors conclusions? There is no analysis here, including statistical analysis, of this potential phenomenon. Please expand on this. Without this, this part of the paper presents little value (since the point of the paper was not, after all, a socio-economic analysis...)

There is a lot of results, and only a little discussion. For example authors wrote about head types, and then end with one sentence “crested heads may provide better protection against the elements in the highland environment, as suggested by previous studies on the adaptive significance of head morphology in chickens”. This is not real discussion. There is no importance, meaning and relevance of results. There are some scattered information among text, but without real meaning and context. I would suggest to distinguish separate section “discussion”.

Section “Correlations of Body Weight and Linear Body Measurement” please correct“…of the study area The Pearson ea revealing…”

“Numerous studies have reported positive correlations between various body measurements in532

indigenous chicken populations, which aligns with the findings of the present study and (79)

examined the phenotypic characterization of indigenous chicken ecotypes in the Central

Highlands of Ethiopia and found significant positive correlations between body weight and other

linear body traits, such as body length (r = 0.44, p < 0.01), body width (r = 0.43, p < 0.01), and

wing span (r = 0.41, p < 0.01)” – too long sentence to understand what authors try to say

Conclusion: “The study found that male chickens (cocks) were heavier than female chickens (hens), indicating the presence of clear sexual dimorphism in the indigenous chicken population” – sexual dimorphism is typical not only for indigenous chicken, but is it is characteristic for this species. Thus there is no real value in this conclusion

I understand that the poor quality of the photos is due to an error at the planning/developing stage (I wrote about it in my first review). Thus, there is no way to correct these photos, but their quality, in my opinion, is not suitable for publication in any journal, especially one with such a as PlosOne. I recommend dropping their publication

**Conclusion**

Please correct citation style in text. Then authors

Please write a discussion that meets the criteria for a valid discussion
