# Supplementary material for: Characterization of indigenous chicken phenotypes in Liban Jawi District, Ethiopia: A qualitative and quantitative analysis
Source: PLoS One. 2025 Jan 10;20(1):e0307793. doi: 10.1371/journal.pone.0307793 (PMC11723647; doi:10.1371/journal.pone.0307793)
Supplement: Attachment — Submitted filename: Response to reviewers.docx [file pone.0307793.s006.docx]

**Response to reviewers' comments (2)**

| **Date:** | Oct 22 2024 01:21PM |
| --- | --- |
| **To:** | "Desalegn Begna" dbegna67@gmail.com |
| **cc:** | lamia_radwan@agr.asu.edu.eg |
| **From:** | "PLOS ONE" plosone@plos.org |
| **Subject:** | PLOS ONE Decision: Revision required [PONE-D-24-28488R2] |
| **Attachment(s):** | [review, chicken plos one.docx](https://www.editorialmanager.com/pone/download.aspx?guid=%7b5fe54a39-6b47-401a-be75-de23e4b56725%7d&scheme=13&id=551826) |

PONE-D-24-28488R2

Characterization of Indigenous Chicken Phenotypes in Liban Jawi District, Ethiopia: A Qualitative and Quantitative Analysis

PLOS ONE

Dear Dr. Desalegn Begna

Thank you for submitting your manuscript to PLOS ONE. After careful consideration, we feel that it has merit but does not fully meet PLOS ONE’s publication criteria as it currently stands. Therefore, we invite you to submit a revised version of the manuscript that addresses the points raised during the review process.

==============================

Dear Dr., Desalegn Begna,

Thank you for submitting your manuscript to PLOS ONE. After careful consideration, we have decided that your manuscript needs Major Revision.

Kind regards,

Prof. Lamiaa Mostafa Radwan, Ph.D.

Academic Editor

PLOS ONE

**Reviewer 1**

There is a lot of progress compared to the first version, however, much still needs to be improved, especially with discussion: Revised to the most possible in our capacity

**Reviewer 2**

Comments and Suggestions
Dear authors, I appreciate your efforts to improve the manuscript; however, some issues remain that require attention.
Esteemed authors, I would be pleased if your manuscript were deemed suitable for publication following the rectification of the following remarks and other reviewer’s comments (If any).
1. It would be advantageous for the problem statement, lines 94-96, to precede the objectives section. We have made this adjustment in the revised version to ensure that the context is clearly established prior to outlining our objectives.
2. Please incorporate references pertaining to the description of the study area. Incorporated
3. Kindly re-evaluate the spelling, grammar, table formatting, and referencing style once more. Done to our level best
4. I remain dissatisfied with Table 2, the PCA, the cluster analysis, and the indices along with the discussion spanning lines 336-342. I would appreciate the inclusion of figures in these multivariate analyses, with morphological indices clearly delineated and analyzed by proposing their functional significance. Done accordingly in the revised document
5. Please refine the conclusions and recommendations section, as the conclusion appears to function merely as a summary. conclusions and recommendations section revised to the bests of our capacity

**Reviewer 3**

Accept

==============================

Please submit your revised manuscript by Dec 06 2024 11:59PM. If you will need more time than this to complete your revisions, please reply to this message or contact the journal office at <a href="mailto:plosone@plos.org">plosone@plos.org. When you're ready to submit your revision, log on to https://www.editorialmanager.com/pone/ and select the 'Submissions Needing Revision' folder to locate your manuscript file.

Please include the following items when submitting your revised manuscript:

- A rebuttal letter that responds to each point raised by the academic editor and reviewer(s). You should upload this letter as a separate file labeled 'Response to Reviewers'.
- A marked-up copy of your manuscript that highlights changes made to the original version. You should upload this as a separate file labeled 'Revised Manuscript with Track Changes'.
- An unmarked version of your revised paper without tracked changes. You should upload this as a separate file labeled 'Manuscript'.

If you would like to make changes to your financial disclosure, please include your updated statement in your cover letter. Guidelines for resubmitting your figure files are available below the reviewer comments at the end of this letter.

If applicable, we recommend that you deposit your laboratory protocols in protocols.io to enhance the reproducibility of your results. Protocols.io assigns your protocol its own identifier (DOI) so that it can be cited independently in the future. For instructions see: <https://journals.plos.org/plosone/s/submission-guidelines#loc-laboratory-protocols>. Additionally, PLOS ONE offers an option for publishing peer-reviewed Lab Protocol articles, which describe protocols hosted on protocols.io. Read more information on sharing protocols at <https://plos.org/protocols?utm_medium=editorial-email&utm_source=authorletters&utm_campaign=protocols>.

We look forward to receiving your revised manuscript.

Kind regards,

Lamiaa Mostafa Radwan, Ph.D.
Academic Editor
PLOS ONE

Additional Editor Comments:

Dear Dr., Desalegn Begna,
Thank you for submitting your manuscript to PLOS ONE. After careful consideration, we have decided that your manuscript needs Major Revision.
Kind regards,
Prof. Lamiaa Mostafa Radwan, Ph.D.
Academic Editor
PLOS ONE

Reviewer 1

There is a lot of progress compared to the first version, however, much still needs to be improved, especially with discussion (Revised an improved to our capacity according to the comment)
Reviewer 2
Comments and Suggestions
Dear authors, I appreciate your efforts to improve the manuscript; however, some issues remain that require attention.
Esteemed authors, I would be pleased if your manuscript were deemed suitable for publication following the rectification of the following remarks and other reviewer’s comments (If any).
1. It would be advantageous for the problem statement, lines 94-96, to precede the objectives section.
2. Please incorporate references pertaining to the description of the study area. Incorporated in the revised document
3. Kindly re-evaluate the spelling, grammar, table formatting, and referencing style once more. All re-evaluated to our capacity as suggested
4. I remain dissatisfied with Table 2, the PCA, the cluster analysis, and the indices along with the discussion spanning lines 336-342. I would appreciate the inclusion of figures in these multivariate analyses, with morphological indices clearly delineated and analyzed by proposing their functional significance. Comments accepted and Figure included, interpreted and discussed with regards to Principal Component Analysis (PCA) of Indigenous Chickens' Morphological Indices
5. Please refine the conclusions and recommendations section, as the conclusion appears to function merely as a summary. Refined as suggested

Reviewer 3
Accept


[Note: HTML markup is below. Please do not edit.]

Reviewers' comments:

Reviewer's Responses to Questions

**Comments to the Author**

1. If the authors have adequately addressed your comments raised in a previous round of review and you feel that this manuscript is now acceptable for publication, you may indicate that here to bypass the “Comments to the Author” section, enter your conflict of interest statement in the “Confidential to Editor” section, and submit your "Accept" recommendation.

Reviewer #1: (No Response)

Reviewer #2: All comments have been addressed

Reviewer #3: All comments have been addressed

2. Is the manuscript technically sound, and do the data support the conclusions?

The manuscript must describe a technically sound piece of scientific research with data that supports the conclusions. Experiments must have been conducted rigorously, with appropriate controls, replication, and sample sizes. The conclusions must be drawn appropriately based on the data presented.

Reviewer #1: No

Reviewer #2: Yes

Reviewer #3: Yes

3. Has the statistical analysis been performed appropriately and rigorously?

Reviewer #1: Yes

Reviewer #2: Yes

Reviewer #3: Yes

4. Have the authors made all data underlying the findings in their manuscript fully available?

The [PLOS Data policy](http://www.plosone.org/static/policies.action#sharing) requires authors to make all data underlying the findings described in their manuscript fully available without restriction, with rare exception (please refer to the Data Availability Statement in the manuscript PDF file). The data should be provided as part of the manuscript or its supporting information, or deposited to a public repository. For example, in addition to summary statistics, the data points behind means, medians and variance measures should be available. If there are restrictions on publicly sharing data—e.g. participant privacy or use of data from a third party—those must be specified.

Reviewer #1: Yes

Reviewer #2: Yes

Reviewer #3: Yes

5. Is the manuscript presented in an intelligible fashion and written in standard English?

PLOS ONE does not copyedit accepted manuscripts, so the language in submitted articles must be clear, correct, and unambiguous. Any typographical or grammatical errors should be corrected at revision, so please note any specific errors here.

Reviewer #1: Yes

Reviewer #2: Yes

Reviewer #3: Yes

6. Review Comments to the Author

Please use the space provided to explain your answers to the questions above. You may also include additional comments for the author, including concerns about dual publication, research ethics, or publication ethics. (Please upload your review as an attachment if it exceeds 20,000 characters)

Reviewer #1: There is a lot of progress compared to the first version, however, much still needs to be improved, especially with discussion

Reviewer #2: Comments and Suggestions
Dear authors, I appreciate your efforts to improve the manuscript; however, some issues remain that require attention.
Esteemed authors, I would be pleased if your manuscript were deemed suitable for publication following the rectification of the following remarks and other reviewer’s comments (If any).
1. It would be advantageous for the problem statement, lines 94-96, to precede the objectives section.
2. Please incorporate references pertaining to the description of the study area.
3. Kindly re-evaluate the spelling, grammar, table formatting, and referencing style once more.
4. I remain dissatisfied with Table 2, the PCA, the cluster analysis, and the indices along with the discussion spanning lines 336-342. I would appreciate the inclusion of figures in these multivariate analyses, with morphological indices clearly delineated and analyzed by proposing their functional significance.
5. Please refine the conclusions and recommendations section, as the conclusion appears to function merely as a summary.

Reviewer #3: (No Response)

7. PLOS authors have the option to publish the peer review history of their article ([what does this mean?](https://journals.plos.org/plosone/s/editorial-and-peer-review-process#loc-peer-review-history)). If published, this will include your full peer review and any attached files.


If you choose “no”, your identity will remain anonymous but your review may still be made public.


**Do you want your identity to be public for this peer review?** For information about this choice, including consent withdrawal, please see our [Privacy Policy](https://www.plos.org/privacy-policy).

Reviewer #1: No

Reviewer #2: No

Reviewer #3: No

[NOTE: If reviewer comments were submitted as an attachment file, they will be attached to this email and accessible via the submission site. Please log into your account, locate the manuscript record, and check for the action link "View Attachments". If this link does not appear, there are no attachment files.]

While revising your submission, please upload your figure files to the Preflight Analysis and Conversion Engine (PACE) digital diagnostic tool, <https://pacev2.apexcovantage.com/>. PACE helps ensure that figures meet PLOS requirements. To use PACE, you must first register as a user. Registration is free. Then, login and navigate to the UPLOAD tab, where you will find detailed instructions on how to use the tool. If you encounter any issues or have any questions when using PACE, please email PLOS at <a href="mailto:figures@plos.org">figures@plos.org. Please note that Supporting Information files do not need this step.

*In compliance with data protection regulations, you may request that we remove your personal registration details at any time.* [*(Remove my information/details)*](https://www.editorialmanager.com/pone/login.asp?a=r)*. Please contact the publication office if you have any questions.*
